# A highly specific antibody against the core fucose of the *N*-glycan in IgG identifies the pulmonary diseases and its regulation by CCL2

**DOI:** 10.1016/j.jbc.2023.105365

**Published:** 2023-10-20

**Authors:** Noriko Kanto, Yuki Ohkawa, Masato Kitano, Kento Maeda, Masafumi Shiida, Tatsuya Ono, Fumi Ota, Yasuhiko Kizuka, Kei Kunimasa, Kazumi Nishino, Mikio Mukai, Masahiro Seike, Arata Azuma, Yoichiro Harada, Tomohiko Fukuda, Jianguo Gu, Naoyuki Taniguchi

**Affiliations:** 1 Depertment of Glyco-Oncology and Medical Biochemistry, Osaka International Cancer Institute, Osaka, Japan; 2 Department of Molecular Biochemistry and Clinical Investigation, Graduate School of Medicine, Osaka University, Osaka, Japan; 3 Research and Development Division, Minaris Medical Co, Ltd, Shizuoka, Japan; 4 Disease Glycomics Team, Global Research Cluster, RIKEN, Saitama, Japan; 5 Institute for Glyco-core Research, Gifu University, Gifu, Japan; 6 Department of Thoracic Oncology, Osaka International Cancer Institute, Osaka, Japan; 7 Deparetment of Medical Check-up, Osaka International Cancer Institute, Osaka, Japan; 8 Department of Pulmonary Medicine and Oncology, Graduate School of Medicine, Nippon Medical School, Tokyo, Japan; 9 Division of Regulatory Glycobiology, Institute of Molecular Biomembrane and Glycobiology, Tohoku Medical and Pharmaceutical University, Sendai, Miyagi, Japan

**Keywords:** N-linked glycosylation, immunoglobulin G (IgG), lung, chemokine, biomarker

## Abstract

Glycan structure is often modulated in disease or predisease states, suggesting that such changes might serve as biomarkers. Here, we generated a monoclonal antibody (mAb) against the core fucose of the *N*-glycan in human IgG. Notably, this mAb can be used in Western blotting and ELISA. ELISA using this mAb revealed a low level of the core fucose of the *N*-glycan in IgG, suggesting that the level of acore fucosylated (noncore fucosylated) IgG was increased in the sera of the patients with lung cancer, chronic obstructive pulmonary disease, and interstitial pneumonia compared to healthy subjects. In a coculture analysis using human lung adenocarcinoma A549 cells and antibody-secreting B cells, the downregulation of the *FUT8* (*α1,6 fucosyltransferase*) gene and a low level of core fucose of the *N*-glycan in IgG in antibody-secreting B cells were observed after coculture. A dramatic alteration in gene expression profiles for cytokines, chemokines, and their receptors were also observed after coculturing, and we found that the identified C-C motif chemokine 2 was partially involved in the downregulation of the *FUT8* gene and the low level of core fucose of the *N*-glycan in IgG in antibody-secreting B cells. We also developed a latex turbidimetric immunoassay using this mAb. These results suggest that communication with C-C motif chemokine 2 between lung cells and antibody-secreting B cells downregulate the level of core fucose of the *N*-glycan in IgG, *i.e.,* the increased level of acore fucosylated (noncore fucosylated) IgG, which would be a novel biomarker for the diagnosis of patients with pulmonary diseases.

Glycosylation is one of the more common posttranslational modifications of proteins (1). *N*- and *O*-glycosylation are ubiquitous and function to regulate the functions of proteins (2). This glycosylation is regulated tightly under the healthy conditions. However, it is well known that unusual glycan structures are often produced in disease or predisease conditions due to the dysregulation of the expression of glycosyltransferases and their donor and acceptor substrates.

These unusual glycans are closely involved in the onset of certain diseases and their progression (3, 4) and have the potential for use as biomarkers (5, 6) and could be used as an indicator for a diagnosis. They would be useful in monitoring the progression and the treatment of the disease (7). Such biomarkers could be identified with a high degree of sensitivity in body fluids; serum, urine, saliva, etc., because obtaining samples of these body fluids is easy and less painful for patients. Human serum contains many biomarker candidates for many diseases including cancers (8). Importantly, more than half of the established cancer biomarkers, for example, alpha-fetoprotein (AFP-L3) (9), CA19-9 (10, 11), etc., are glycoconjugates (12). This suggests that targeting cancer-related glycans would be a reasonable strategy for developing novel biomarkers for cancer patients. Focusing on *N*-glycans, it was reported that the core fucose, catalyzed by the action of FUT8 (α1,6 fucosyltransferase), is highly associated with various diseases in relation to specified targeted molecules (6, 13).

Immunoglobulin G (IgG) is a protein that is present in relatively high levels in human serum (14). Notably, human IgG carries an *N*-glycan chain attached to a conserved glycosylation site at Asn-297 in its constant heavy 2 (CH2) domain (15). This glycan structure would be an ideal research target for the following two reasons: (1) purifying IgG from serum is simple and easy and (2) only one *N*-glycan chain exists in the CH2 domain of IgG, which allows the complexity of the structural analysis of glycans to be avoided. An excellent overview regarding this appeared recently (16). Interestingly, it was noted that the glycan structure of IgG, especially the core fucose, is significantly changed in disease or predisease states. Over 90% of IgG in sera generally carries a core fucose (17) but it can be dramatically downregulated, for example, in autoimmune thyroid diseases (18) and infectious diseases including human immunodeficiency virus (19) and dengue virus (20). Very recently, it was also reported that the severity of COVID-19 infections was highly associated with increased levels of afucosylated (nonfucosylated) IgG against the COVID-19 spike protein (21, 22). The level of the core fucose of IgG is also associated with aging (23), colorectal cancer (24), rheumatoid arthritis (25), hemolytic diseases (26), thrombocytopenia (27), systemic lupus erythematosus (28) and related diseases. A review paper describing the function of core fucosylation in B cell receptor and IgG was also published (29). These reports strongly suggest that the core fucose of the *N*-glycan in IgG is important for the diagnosis and monitoring of the disease state. For these reasons, the core fucose of the *N*-glycan in IgG is also the focus of interest in the drug discovery field for developing antibody-based drugs. The deletion of the core fucose of IgG, *i.e.,* an increase in acore fucosylated (noncore fucosylated) IgG, dramatically enhances antibody-dependent cellular cytotoxicity by strengthening its affinity for the FcγIIIa receptor that is expressed on myeloid cells and natural killer cells (30). Given the above information, we generated a novel monoclonal antibody against core fucosylated human IgG. Because this antibody does not recognize acore fucosylated (noncore fucosylated) human IgG, therefore, the level of core fucosylated IgG can be easily analyzed in a straightforward manner. Moreover, the core fucose of the therapeutic IgG antibodies against cancer that are used today can also be easily analyzed and monitored.

In this study, we report on the specificity of this antibody and the availability for Western blotting and enzyme-linked immunosorbent assay (ELISA) for measuring the usual natural IgG antibodies. We observed low levels of the core fucose of IgG, namely increased levels of acore fucosylated (noncore fucosylated) IgG, in sera in lung cancer patients, as well as patients with chronic obstructive pulmonary disease (COPD) and interstitial pneumonia (IP). Based on these findings, we propose a mechanism by which the core fucose level of IgG is downregulated in antibody-secreting B cells.

## Results

### Recognition specificity of the antibody against core fucose of human IgG

**Figure 1:**
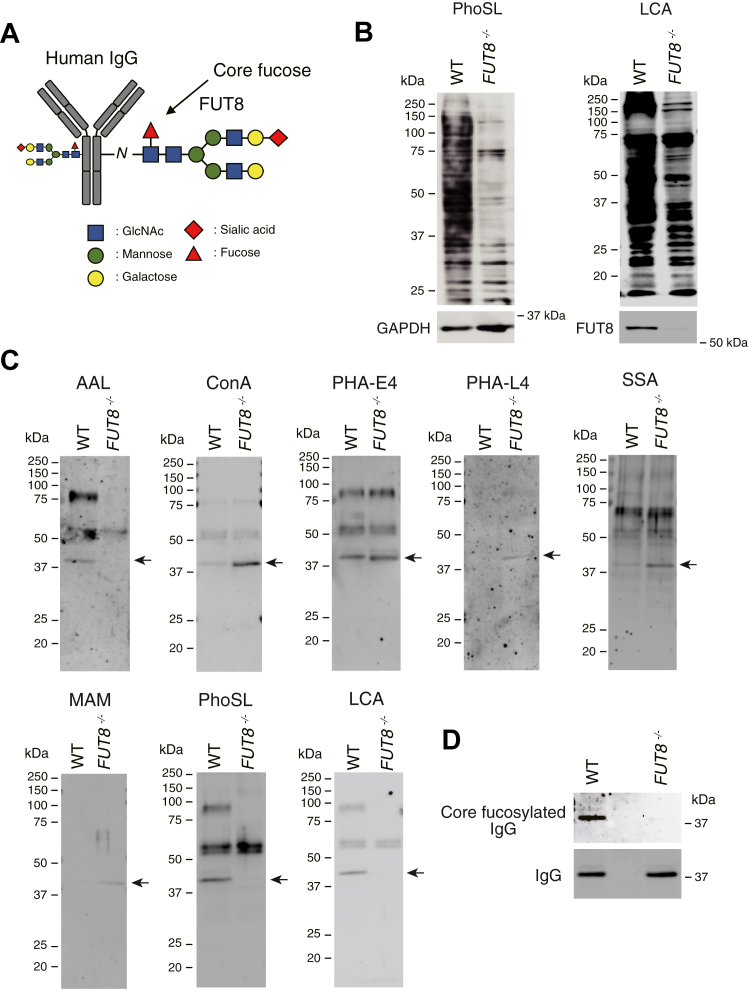
**Knockout of the *Fut8* gene disrupts the reactivity of the antibody against core fucose of *N*-glycan in IgG.***A*, representative *N*-glycan structure in human IgG. FUT8 (α1,6 fucosyltransferase) is an enzyme to generate the core fucose of *N*-glycan. *B*, PhoSL and LCA lectin blotting in *Fut8*-deficient HEK293 cells (*Fut8*^*−/−*^) and wildtype cells (WT). Total cell lysates (20 μg) were analyzed, and GAPDH was used as a loading control. The protein expression of FUT8 was also analyzed with an anti-FUT8 antibody. *C*, lectin blotting of IgGs derived from *Fut8*-deficient HEK293 cells (*Fut8*^*−/−*^) and wildtype cells (WT). After transfection with an IgG expression vector (human IgG_1_ CH1/CH2/CH3 portion), the secreted IgGs in the culture media were purified and enriched with ProteinG/Sepharose. A part of those samples was analyzed with lectins (AAL, ConA, PHA-E4, PHA-L4, SSA, MAM, PhoSL, and LCA). *Arrows* indicate the CH1/CH2/CH3 portion of human IgG_1_. *D*, Western blotting of IgGs. Enriched IgGs used in *C* were also analyzed with an antibody against the core fucose of *N*-glycan in IgG and an anti-human IgG antibody. AAL, *Aleuria aurantia* lectin; CH2, constant heavy 2; FUT8, α1,6 fucosyltransferase; IgG, immunoglobulin G; LCA, *Lens culinaris agglutinin* lectin; mAb, monoclonal antibody; MAM, *Maackia amurensis* lectin; PhoSL, *Pholiota squarrosa* lectin; SSA, *Sambucus sieboldiana* lectin.

FUT8 is the sole enzyme responsible for generating the core fucose of *N*-glycans (Fig. 1*A*) (31, 32, 33). To demonstrate the antibody specificity against core fucose of human IgG, we examined the human IgG_1_ generated in *FUT8* gene-knockout HEK293 cells (*FUT8*^*−/−*^). As shown in Figure 1*B*, the core fucose that was detectable in *FUT8*^*−/−*^ cells with PhoSL (*Pholiota squarrosa* lectin) and LCA (*Lens culinaris agglutinin* lectin) in Lectin blotting compared to wildtype cells (*FUT8*^*+/+*^) was dramatically decreased. Using these *FUT8*^*−/−*^ and wildtype cells, we overexpressed human IgG_1_ and analyzed the glycan structure of the molecule with lectins and the antibody against core fucose of human IgG. Lectin blotting with AAL (*Aleuria aurantia* lectin), PhoSL, and LCA confirmed the lack of core fucose in the *N*-glycan of IgG_1_ that was derived from *FUT8*^*−/−*^ cells (Fig. 1*C*). Using the same samples, we performed Western blotting and observed the specificity of the antibody against the core fucose of human IgG_1_ (Fig. 1*D*). We also performed further experiments to confirm the specificity of this antibody for recognizing its target. The intermolecular interaction assay based on surface plasmon resonance also demonstrated that the specificity of this antibody for recognizing its target did not depend on IgG subclasses (Fig. S1, *A* and *B*). In ELISA, the antibody could detect human IgG_4_ generated in wildtype of CHO cells (*Fut8*^*+/+*^), whereas no binding was observed in *Fut8* gene-knockout CHO cells (*Fut8*^*−/−*^) (Fig. S2, *A*–*C*). Importantly, this antibody did not recognize the core fucose in other types of immunoglobulins, for example, human IgA, human IgM, and rabbit IgG as well as nonrelevant glycoproteins such as human lactoferrin and bovine thyroglobulin containing core fucose structure (Figs. S2*D* and S3, *A*, and *B*). We additionally analyzed the human pool serum by Western blotting with this antibody and observed a single band indicating IgG heavy chain carrying core fucose (Fig. S4). These data surely demonstrate the specificity of the antibody against core fucose of the *N*-glycan in human IgG.

### ELISA indicated low core fucose levels of IgG in the sera of patients with lung cancer, COPD, and IP

**Figure 2:**
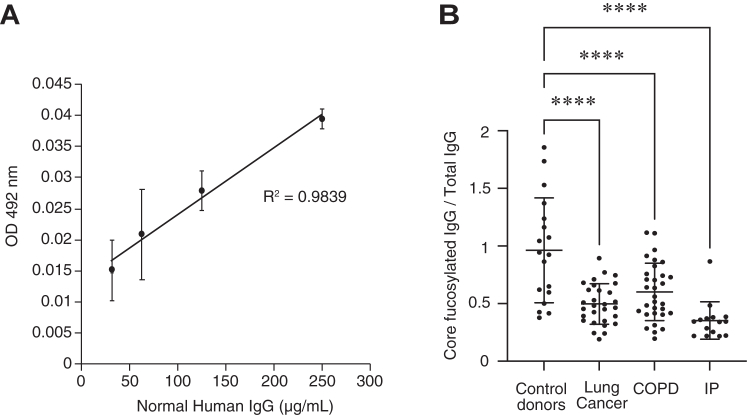
**Low level of core fucosylation of IgG in sera of the patients with pulmonary diseases.***A*, a typical standard curve for ELISA for purified normal human IgG using the antibody against core fucose of *N*-glycan in IgG. *B*, ELISA using the antibody against the core fucose of the *N*-glycan in IgG to measure the core fucosylation level of IgGs in sera of lung cancer, chronic obstructive pulmonary disease (COPD), interstitial pneumonia (IP) patients, and control healthy donors. The ratios of core fucosylated IgG against total IgG were plotted. Lung cancer: N = 29, COPD: N = 31, IP: N = 15, healthy donors: N = 18. ∗∗∗∗*p* < 0.0001. IgG, immunoglobulin G.

The terminal sialylation level of the *N*-glycan in IgGs sometimes reflects a disease state. For example, a low level of sialylation of IgG was reported in autoimmune diseases, while the levels of sialylation of IgG was high in cancer patients (15). In addition, it was reported that the terminal sialylation level of *N*-glycan in IgG can serve as a biomarker for chronic inflammatory demyelinating polyneuropathy (34) and cardiovascular disease (35). Based on these reports, we hypothesized that the core fucose level of the *N*-glycan of IgGs would also be altered in a disease or a predisease state. To answer this question, we assessed the level of core fucose of IgG in sera of patients with lung cancer, COPD, IP, and control healthy donors by ELISA with the antibody against the core fucose of *N*-glycan in IgG. We first assayed the level of core fucose of purified normal human IgG in a dose-dependent manner and confirmed the availability of this antibody for ELISA (Fig. 2*A*). We then performed ELISA again and found low levels of the core fucose of IgG in patients with lung cancer, COPD, and IP, compared to control healthy donors (Fig. 2*B*). We also assessed the terminal sialylation levels of the *N*-glycan in IgGs in the same subjects with *Sambucus sieboldiana* lectin and *Maackia amurensis* lectin by lectin blotting. Notably, the terminal sialylation levels were similar between all types of patients (Fig. S5), indicating that the terminal sialylation of IgG is not important for making a diagnosis. In addition, KL-6 was established as a useful serum biomarker for IP patients (36). Concerning this, we assayed KL-6 again in the same subjects, but we did not observe any relationships between the level of KL-6 and the core fucosylation of IgG (Fig. S6). These data suggest that the low core fucose levels of IgG in sera that were detected with this antibody make it an ideal biomarker candidate for identifying patients with pulmonary diseases.

### Cocultures with lung cancer cells decreased *FUT8* expression and the level of core fucose of IgG in antibody-secreting B cells

**Figure 3:**
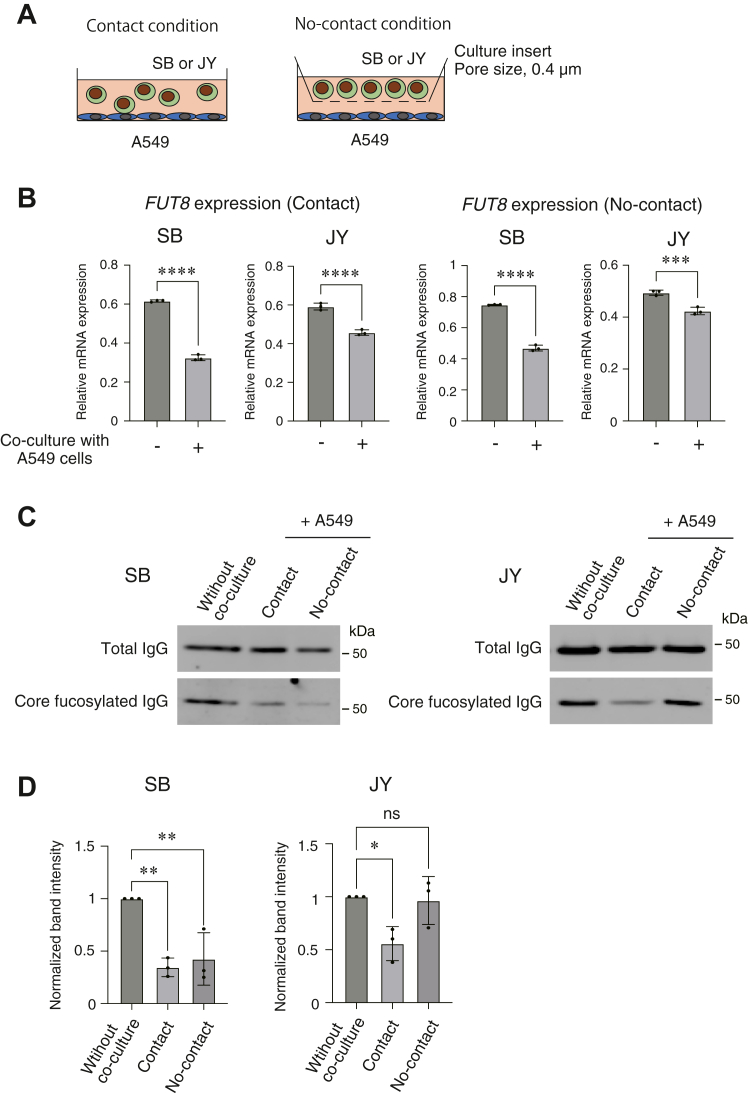
**Coculturing with lung cancer cells downregulates the *Fut8* gene and the level of core fucose of *N*-glycan in IgG in antibody-secreting B cells.***A*, scheme for the coculture analyses under conditions of contact or no-contact using human lung adenocarcinoma A549 cells and antibody-secreting B cells, SB and JY. A549 is an adherent cell, while SB and JY cells are nonadherent cells. *B*, *FUT8* gene expression was examined by RT-qPCR in antibody-secreting SB and JY cells after coculture with A549 cells on the condition of contact or no-contact for 3 days. Expressions were normalized to the *GAPDH* gene. ∗∗∗*p* < 0.005, ∗∗∗∗*p* < 0.001. *C*, the level of core fucose of *N*-glycan in IgGs in SB and JY cells after coculturing with A549 cells. Secreted IgGs into the culture media were enriched with ProteinG/Sepharose and examined by Western blotting using an antibody against the core fucose of *N*-glycan in IgG and an anti-human IgG antibody. The values under the images indicate the normalized band intensities of the level of core fucose of *N*-glycan in IgG in SB and JY cells after coculture with A549 cells relative to those without coculture. *D*, the band intensities in *C*. Three independent experiments were performed, and the band intensities normalized to the condition without coculture were measured. ∗*p* < 0.05, ∗∗*p* < 0.01. FUT8, α1,6 fucosyltransferase; IgG, immunoglobulin G; ns, not significant; RT-qPCR, reverse transcription-quantitative polymerase chain reaction.

The expression of the *FUT8* gene is required to generate the core fucose of the *N*-glycan in IgG (Fig. 1). To address the issue of why the core fucose level of IgG decreases in the sera of lung cancer patients and whether the expression of the *FUT8* gene is involved in this, we next performed coculture analyses using human lung adenocarcinoma cell line A549 and human B cell lymphoma cell lines. Before performing the coculture analyses, we measured IgG expression in five different human B cell lymphoma cell lines, SB, Raji, p32/ISH, JY, and Ramos, and found that the SB and JY cells expressed IgGs as well as core fucosylated IgGs (Fig. S7). Therefore, we used SB and JY cells as antibody-secreting B cells. Using A549, SB, and JY cells, we performed coculture analyses under two conditions: contact or no-contact, as illustrated in Figure 3*A*. Interestingly, after coculture for 3 days, the gene expression of *FUT8* was significantly decreased in SB and JY cells under both conditions (Fig. 3*B*). The level of core fucose of the *N*-glycan in IgG was also decreased (Fig. 3*C*). These consistent data indicate that the downregulation of the *FUT8* gene in antibody-secreting B cells is induced by coculturing with lung cancer cells, which results in a decrease in the level of core fucose of the *N*-glycan in IgG. Notably, incubation with conditioned media prepared from A549 cells failed to downregulate the *FUT8* gene in SB and JY cells, which strongly suggests that coculturing is required to downregulate the *FUT8* gene in these cells (Fig. S8).

### Coculturing of lung cancer cells with antibody-secreting B cells altered gene expression profiles

**Figure 4:**
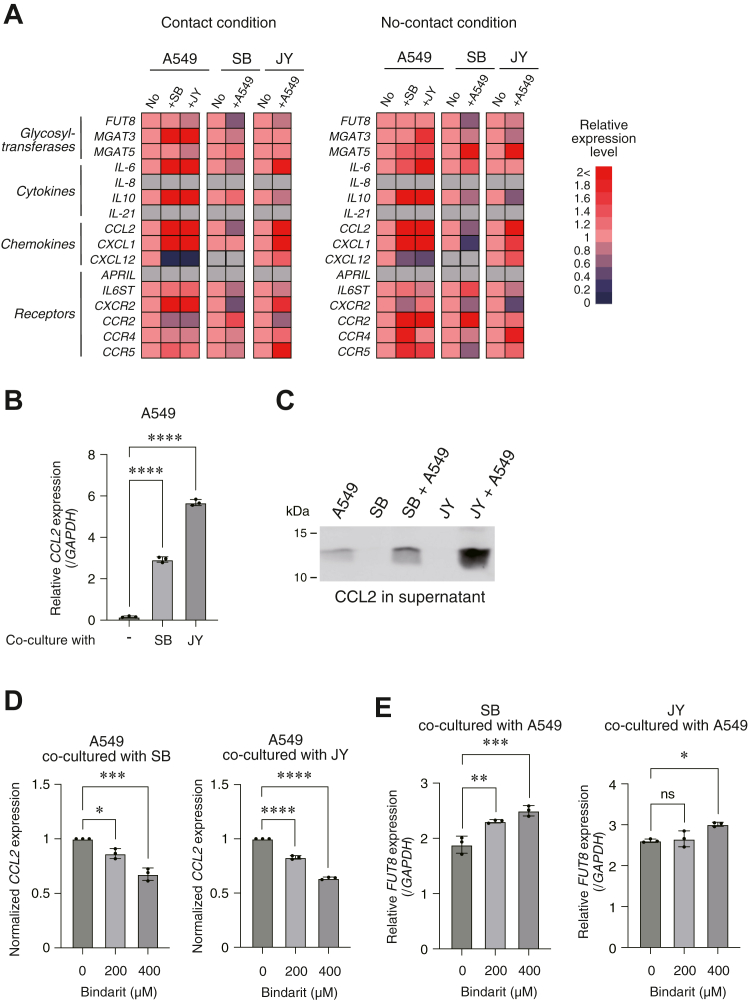
**Gene expression profiles in A549 and antibody-secreting B cells after coculturing.***A*, heatmaps of gene expression examined by RT-qPCR in A549, SB, and JY cells after coculture for 3 days under conditions of contact or no-contact. Gene expression levels in the cells after coculturing relative to the cells without co∗-culture are shown. *Gray color* indicates no expression. *B*, *CCL2* gene expression in A549 cells after coculture with SB or JB cells on the condition of contact examined by RT-qPCR. Expressions were normalized to the *GAPDH* gene. *C,* CCL2 protein expression in supernatant examined by Western blotting. After coculture SB and JB cells with or without A549 cells on the condition of contact, culture supernatant was collected and concentrated. An equally part of resulting samples was analyzed. *D, CCL2* gene expression in A549 cells under the presence of Bindarit, an inhibitor of *CCL2* expression, was examined by RT-qPCR. Expressions were normalized to the condition without Bindarit treatment (0 μM). *E, FUT8* gene expression in SB and JY cells after coculture with A549 under the presence or absence of Bindarit. Expressions were examined by RT-qPCR and normalized to the *GAPDH* gene. ∗*p* < 0.05, ∗∗*p* < 0.01, ∗∗∗*p* < 0.005, ∗∗∗∗*p* < 0.001. CCL2, C-C motif chemokine 2; FUT8, α1,6 fucosyltransferase; ns: not significant; RT-qPCR, reverse transcription-quantitative polymerase chain reaction.

Next, to examine the mechanisms responsible for the downregulation of the *FUT8* gene in antibody-secreting B cells, we analyzed the gene expression profiles in these cells after coculture by means of the reverse transcription-quantitative polymerase chain reaction (RT-qPCR). As shown in Figure 3*B*, the coculture under conditions of no-contact resulted in a decreased *FUT8* gene expression in antibody-secreting B cells, indicating that the downregulation of *FUT8* was induced by secreted molecules. For this reason, we focused on typically secreted molecules such as cytokines, chemokines, and their receptors. As shown in Figure 4*A*, the gene expression of *interleukin-6* (*IL-6*), *interleukin-10*, *C-C motif chemokine 2* (*CCL2*), and *C-X-C motif chemokine 1* (*CXCL1*) in A549 were induced strongly after coculture under both conditions of contact and no-contact. No similar gene expression profiles for SB and JY after coculture under both conditions. If cytokines or chemokines that are upregulated in A549 mediate the downregulation of the *FUT8* gene in SB and JY cells, the expression of their receptors: *interleukin-6 receptor subunit beta*, *C-X-C motif chemokine receptor 2*, *CC chemokine receptors* (*CCRs*), should be sufficiently detectable in these cells. In a RT-qPCR analysis, the gene expression of *interleukin-6 receptor subunit beta*, *CCR2*, and *CCR4* were detectable in both SB and JY cells after coculturing under both conditions of contact and no-contact (Fig. 4*A*). On the other hand, the gene expression of *C-X-C motif chemokine receptor 2*: a receptor for CXCL1, was substantially decreased after coculturing especially in SB cells under conditions of contact and in JY cells under conditions of no-contact (Fig. 4*A*). This indicates that CXCL1 is not a functional molecule in this case. These collective findings indicate that that IL-6 and CCL2 are potential candidates for mediation of the downregulation of the *FUT8* gene in antibody-secreting B cells.

### Inhibition of *CCL2* expression upregulated *FUT8* gene in antibody-secreting B cells

In A549 cells, a dramatic induction of the gene and protein of CCL2 were observed after coculturing with SB and JY cells under conditions of contact, as shown in Figure 4, *B* and *C*. To address the issue of whether CCL2 is involved in the downregulation of *FUT8*, we performed coculture analyses a second time in the presence of an inhibitor against *CCL2* gene expression, Bindarit (37, 38). As shown in Figure 4*D*, incubation with Bindarit significantly decreased *CCL2* gene expression in A549 cells after coculturing with SB and JY cells, and this decrease was dose dependent. Under the same conditions, *FUT8* gene expression was upregulated in SB and JY cells, especially at a higher concentration of Bindarit (400 μM) (Fig. 4*E*). These data suggest that CCL2 was one of the mediators of the downregulation of the *FUT8* gene in antibody-secreting B cells. This indicates that the CCL2 expressed in cancer cells potentially decreases the level of core fucose of the *N*-glycan in IgG in sera of patients with lung cancer.

### The latex turbidimetric immunoassay can be used to identify patients with pulmonary diseases

**Figure 5:**
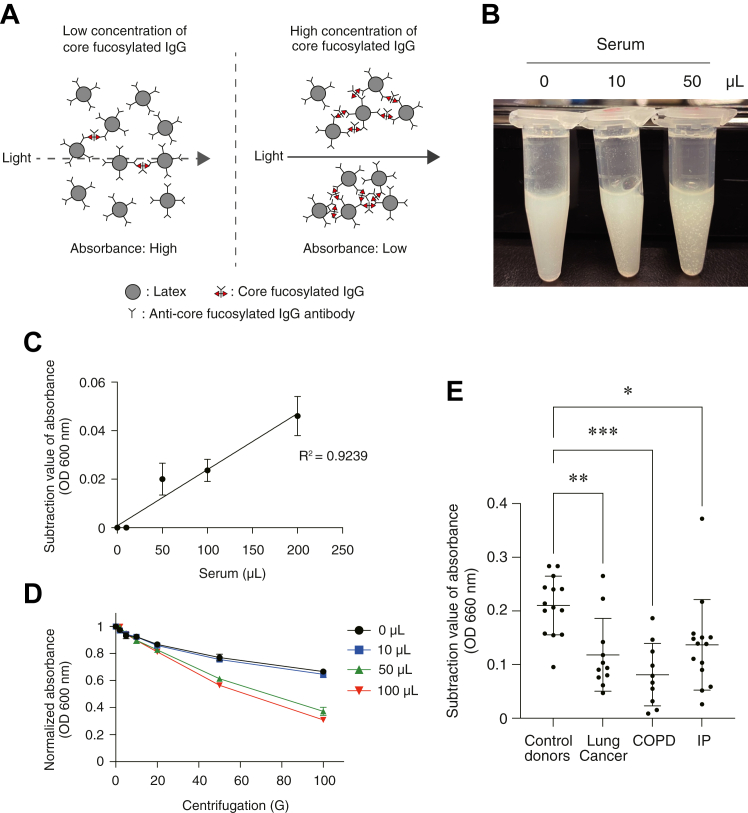
**The latex turbidimetric immunoassay can be used to identify patients with pulmonary diseases.***A*, scheme for the latex turbidimetric immunoassay using the antibody against core fucose of *N*-glycan in IgG. The aggregation of latex beads coated with the antibody can be observed in a dose-dependent manner. A lower absorbance value indicates a higher concentration of core fucose of *N*-glycan in IgG in the subjects. *B*, an image of aggregated latex beads. Human serum from a control healthy donor (0, 10, or 50 μl) was incubated with the solution of latex beads coated with the antibody against core fucose of *N*-glycan in IgG. *C*, a typical standard curve for the latex turbidimetric immunoassay. The human pooled serum (0, 10, 50, 100, or 200 μl) was analyzed, and the absorbance values (600 nm) were subtracted from those just after the addition of sera (time = 0). *D*, the absorbance of the solution of latex beads after centrifugation. After incubation with human pooled serum (0, 10, 50, or 100 μl), the latex solution was centrifugated at 2, 5, 10, 20, 50, or 100 G for 20 s, then, the absorbance of the solution was analyzed. The absorbance values (600 nm) were normalized to those without centrifugation. *E*, the absorbance of the solution of latex beads after incubation with the sera of the patients with lung cancer (N = 11), chronic obstructive pulmonary disease (COPD) (N = 10), interstitial pneumonia (IP) (N = 14), and control healthy donors (N = 14). The absorbance values (660 nm) were subtracted from those just after the addition of sera (time = 0). ∗*p* < 0.05, ∗∗*p* < 0.01, ∗∗∗*p* < 0.001. IgG, immunoglobulin G.

The levels of core fucose of IgG in sera of patients with pulmonary diseases were significantly lower than those of control healthy donors, as determined by ELISA as shown in Figure 2*B*. To confirm this, we developed another detection system using latex that is coated with an antibody against the core fucose of the *N*-glycan in the IgG antibody. When the sample contains a core fucosylated IgG, the latex induces agglutination, which results in a decreased absorbance value (Fig. 5*A*). As shown in Figure 5, *B* and *C*, we observed latex agglutination after mixing the latex with sera, and the values for the subtraction of absorbance were increased linearly in a dose-dependent manner. The agglutinated latex can be removed by centrifugation; hence, the absorbance values were decreased after centrifugation when the latex was mixed with sera (Fig. 5*D*). Using this system, we tested the sera of lung cancer, COPD, IP patients, and healthy control donors and found that latex agglutination was stronger in control healthy donors than in patients with lung cancer, COPD, or IP. The bigger values for the subtraction of absorbance reflected a stronger latex agglutination in the sera of healthy control donors (Fig. 5*E*). These data demonstrated again the conclusion that low core fucose levels of *N*-glycan in IgG in sera would be a biomarker candidate for patients with pulmonary diseases such as lung cancer, COPD, and IP.

## Discussion

The structure of the *N*-glycan in IgG is thought to be a key molecule for the development of biomarkers and antibody-based drugs (39). Among diverse *N*-glycan structures, targeting the core fucose is suitable because the generation of its glycan structure is regulated by one specific glycosyltransferase, FUT8. Intriguingly, in this study, we report that the core fucose of the *N*-glycan in IgG in sera is measurable lower in patients with pulmonary diseases such as lung cancer, COPD, and IP. This is the first report of the use of a novel monoclonal antibody against the core fucose of the *N*-glycan in IgG and points to the possible utility of the core fucose of *N*-glycan in IgG as a new biomarker for patients with pulmonary diseases. The importance of analyzing the level of the core fucose of the *N*-glycan in IgG has increased with passing years (40). The biggest impact was the report that the core fucose level in IgG is decreased in COVID-19 patients (21, 22). Notably, almost all of the IgG molecules against SARS-CoV-2 lack the core fucose. A research group also reported that the core fucose of the *N*-glycan in IgG is an endogenous ligand for Dectin-1 (41). These results suggest the potential for using this structure for the diagnosis of certain diseases, and the close relationship between the core fucosylation of IgG and inflammation is a valuable indicator.

B cells are produced by hematopoietic stem cells in the bone marrow and differentiate into plasma cells/antibody-secreting B cells in the spleen and peripheral tissues such as lymph nodes (42). In the case of an infection, plasma cells secrete pathogen-specific antibodies to eliminate pathogens and their harmful agents (43). The platform for the maturation of the adaptive immune system is located in the spleen and lymph nodes. Hence, the majority of plasma cells are located in those tissues; however, it is interesting to note that a fraction of plasma cells are present in the blood as well as in the cancer tissues (44). B cells infiltrate into breast cancer tissues and secrete antibodies at that location, which contributes to the development of antitumor effects (45). A high density of B cells and plasma cells in adenocarcinomas of the esophagogastric junction is an index for a good prognosis (46). In addition, a recent review paper summarized the state of our knowledge regarding the prognostic significance of tumor-infiltrating B cells and plasma cells in human cancers (47). These reports strongly indicate that cancer cells are communicating with B cells and/or plasma cells in a tumor-microenvironment, which potentially affects the secretion of IgG as well as the glycan structure of the IgG in antibody-secreting B cells. Our results reported in this study confirm that the communication between cancer cells and antibody-secreting B cells results in the downregulation of the *FUT8* gene and the level of the core fucose of the *N*-glycan in IgG in antibody-secreting B cells.

The *N*-glycan structure is sometimes modulated by proinflammatory cytokines such as IL-6 and tumor necrosis factor-alpha. These cytokines change the expression patterns of glycosyltransferases and sulfotransferases at the transcriptional level, leading to the production of sialylated structures such as sialyl-Lewis^X^ (48, 49). It was also reported that tumor necrosis factor-alpha modulates *N*-glycan structure through the downregulation of several different *N*-acetylglucosaminyltransferases (*MGAT1*, *MGAT2*, *MGAT3*, *MGAT4B*, and *MGAT5B*) in ocular autoimmune disease (50). The physiological mediators for the suppression of the *FUT8* gene have not been identified well at this time, although we found that CCL2 was a potential suppressor of the *FUT8* gene as demonstrated in coculture analyses in this study. Notably, CCL2 is overexpressed in some cancers: lung cancer (51), renal cancer (52), etc. This overexpression was reported to be associated with tumor malignancy. Therefore, the inhibition of CCL2 leads to weakened malignant phenotypes and an increased sensitivity to treatment (53, 54). Unfortunately, the detailed mechanisms by which CCL2 suppresses *FUT8* gene expression have not yet been revealed. This represents an important point that needs to be investigated future research.

The structural changes in the *N*-glycan of IgG would be an attractive index for diagnosis; however, a mass spectrometry analysis is required to provide a convincing analysis of a *N*-glycan structure. Mass spectrometry analysis is a complex methodology for nonspecialists in clinical or research in different fields. Using lectins such as AAL (55), PhoSL (56), or LCA (57) is another way to analyze the *N*-glycan structure of IgG; however, these lectins can recognize a broad range of *N*-glycan structures including core fucose. Very recently, a molecule that recognizes IgG Fc glycoforms was generated using nanobody technology and was used to measure afucosylated (nonfucosylated) IgG_1_ in sera of SARS-CoV-2–infected patients and patients infected with the dengue virus (58). Concerning this, the novel antibody against the core fucose of *N*-glycan in IgG that was developed in this study would be a powerful tool for overcoming the hindrances associated with the above methods. We hope that this novel antibody will allow the early detection of pulmonary diseases in patients with pulmonary diseases.

## Experimental procedures

### Generation of an antibody against core fucose of *N*-glycan in IgG (an anti-core fucosylated IgG antibody)

The mouse monoclonal antibody against core fucose of *N*-glycan in IgG (an anti-core fucosylated IgG antibody) by immunizing FLAG-tagged human IgG_4_ Fc protein (50 μg) to *Fut8*-knockout mice (6 weeks old) was produced in collaboration between our laboratory and the Minaris Medical Co, Ltd (Tokyo) (International Patent No. WO2018/052041). Booster immunization with 2 mg of aluminum gel (Wako) and the whole-cell pertussis vaccine (1 × 10^9^ cells) (Serum Research Institute) was also performed. After immunizing four times (once a week), splenocytes isolated from the immunized mice were fused with mouse myeloma P3U1 cells by using PEG1500 (Roche). The antibodies derived from the clonal hybridomas were tested with recombinant FLAG-tagged human IgG_4_ generated in *Fut8*-knockout CHO cells or parental CHO cells by ELISA. The *Fut8*-knockout mouse was previously established in our research group (59, 60). The hybridoma was cultured in GIT medium (Wako), and the antibodies secreted from the cells were collected and purified by using *PROTEUS* Protein G Midi Kit (M&S TechnoSytems Inc) following a manufacturer’s protocol. A part of the purified antibody was labeled with HRP by using a Peroxidase Labeling Kit-NH2 (Dojindo Molecular Technologies, Inc).

### Collection of human serum samples

A few milliliters of whole blood were drawn from patients with lung cancer, COPD, IP, and control healthy donors at Osaka International Cancer Institute and Nippon Medical School Hospital. The whole blood was collected into a test tube with a coagulation activator and gel serum separator and incubated for 30 min at room temperature. After incubation, the tube was centrifuged at 2095*g* for 5 min. The supernatant was collected as a serum sample.

### Cell and cell culture

Human embryonic kidney cells HEK293 and human adenocarcinoma cell line A549 were obtained from ATCC (American Type Culture Collection). The *FUT8* gene-knockout HEK293 cell (*FUT8*^*−/−*^) was established in the previous study (61). Human B cell lymphoma cell lines SB, p32/ISH, and Ramos were obtained from JCRB (Japanese Collection of Research Bioresources) Cell Bank. Raji and JY were obtained from RIKEN Bioresource Center and ECACC (European Collection of Authenticated Cell Cultures), respectively. HEK293 and A549 were cultured in DMEM (Wako), while B cell lymphoma cell lines were cultured in RPMI1640 (Wako) supplemented with 10% fetal bovine serum and 1% penicillin–streptomycin (Thermo Fisher Scientific) under 5% CO_2_ at 37 °C.

### Preparation of cell lysates

Cells were washed three times with ice-cold PBS and lysed with cell lysis buffer (1% NP-40 in TBS) including protease inhibitors (Roche). The lysates were sonicated briefly and then centrifuged at 15,000*g* at 4 °C for 5 min to remove insoluble materials. The protein concentration of the lysate was analyzed with BCA protein assay kit (Bio-Rad).

### Purification of IgG from sera and cell culture media

The 20 μl of ProteinG/Sepharose (GE Healthcare) was added to sera diluted 100 times diluted with cell lysis buffer (1% NP-40 in TBS) or 10 ml of cell culture media and incubated at 4 °C overnight under rotation. After washing three times with ice-cold cell lysis buffer, IgG bound to ProteinG/Sepharose was eluted by boiling for 5 min in sample buffer (50 mM Tris (pH6.8), 2% (w/v) SDS, 2.5% (v/v) 2-mercaptoethanol, and 9% (v/v) glycerol). These samples were applied to Lectin blotting and Western blotting.

### Lectin blotting and Western blotting

Cell lysates and purified IgGs were separated by SDS-PAGE using 10% acrylamide gels. After transferring onto the nitrocellulose membrane, the membrane was blocked with 2% BSA in TBST (0.1% Tween20 in TBS) and incubated with lectins (AAL, ConA, PHA-E4, PHA-L4, *Sambucus sieboldiana* lectin, *Maackia amurensis* lectin, PhoSL, and LCA) (MGC Woodchem Corp), an anti-core fucosylated antibody or an anti-human IgG antibody. An anti-FUT8 antibody was generated in our laboratory. An anti-CCL2 antibody was obtained from Abcam (Cambridge). For lectin blotting, the membrane was analyzed by using VECTASTAIN ABC Standard Kit (Vector Labs). For Western blotting, the membrane was incubated with an anti-mouse IgG antibody labeled with HRP or an anti-human IgG antibody labeled with HRP (GE Healthcare). To visualize the bands, we used Western Lightning Plus-ECL (PerkinElmer) and ImageQuant LAS-4000mini (Cytiva).

### Enzyme-linked immunosorbent assay

ELISA was performed with minor modifications as described previously (62). In brief, 0.5 μg of the antibody against core fucose of *N*-glycan in IgG diluted in 100 mM NaCO_3_ (pH 9.6) was added onto the 96-well plates (Sumitomo Bakelite Co, Ltd) and incubated at 4 °C overnight. After washing three times with washing buffer (0.05% Tween20 in PBS), 5% BSA (Sigma-Aldrich) in PBS was added, and the sample incubated for 1 h at room temperature. After washing three times with washing buffer again, 100 μl/well of sera diluted in PBS (1:100 or 1:200) was added and incubated for 2 h at room temperature. The wells were then washed five times and incubated with an anti-human IgG antibody labeled with HRP (GE Healthcare) (1:2000). After incubation for 1 h at room temperature, the wells were washed five times, and the substrate solution (0.26 mg/ml of *o*-Phenylenediamine in 0.1 M citrate buffer (pH 5.0) including 0.009% H_2_O_2_) was added. After incubation for 10 min in the dark, the absorbance values at 492 nm were measured with a plate reader, Infinite M Plex (TECAN).

### Reverse transcription-quantitative polymerase chain reaction

RNA extraction from cells was performed with a RNeasy Mini Kit (Qiagen) following the manufacturer’s protocol. Reverse transcription was performed with SuperScript IV Reverse Transcriptase (Invitrogen) and dT (20) primer following the manufacturer’s protocol. Real-time PCR was performed with THUNDERBIRD Next SYBR qPCR Mix (TOYOBO) and analyzed with 7500 Real-Time PCR System (Applied Biosystems). Primers used in this study are listed in Table S1.

### Coculture analyses

A549 cells (5 × 10^5^) and SB or JY cells (5 × 10^5^) were cultured in 6-well plates (IWAKI) with RPMI1640 supplemented with 10% fetal bovine serum and 1% penicillin-streptomycin. On the condition of no-contact, cells were separated with Polycarbonate Cell Culture Insert (pore size, 0.4 μm) (Thermo Fisher Scientific). In the *CCL2* inhibition assay, A549 cells were preincubated overnight with 200 μM or 400 μM of Bindarit (Selleck Chemicals), then coculture was started for 3 days under the presence of 200 μM or 400 μM of Bindarit freshly in the media.

### Latex turbidimetric immunoassay

The latex (particle size, 200 nm) was coated with an antibody against the core fucose of *N*-glycan in IgG (an anti-core fucosylated IgG antibody) following the manufacturer’s protocol (Fujikura Kasei). Briefly, 1 ml of 0.5% latex solution in 10 mM Hepes (pH 7.0) was incubated with 300 μl of 1 mg/ml WSC (Water Soluble Carbodiimide) (Wako). After incubation for 20 min under rotation, 100 μg of anti-core fucosylated IgG was added and incubated for 1 h. A 15 μl portion of 10% Tween20 was then added, and the sample was then ultrasonicated for 10 min. Centrifugation at 15,000*g* for 30 min was performed to remove the solution and added 1 ml of blocking buffer (1% BSA) to the latex. Centrifugation was performed again after incubation and ultrasonication. Then, the latex was resuspended in 1 ml of 0.1% BSA in 20 mM Hepes (pH 7.0). The 200 μl of the latex solution was also diluted with 750 μl of 0.1% BSA in 20 mM Hepes (pH 7.0) and mixed 50 μl of serum. After incubation at room temperature overnight under rotation, 100 μl of solution was analyzed with a plate reader, Infinite M Plex. The absorbance values at 600 nm or 660 nm were measured.

### Statistical analysis

All statistics were performed using Graph Pad Prism 9.0 software (Graph Pad Software). All data are presented as mean ± standard deviation (SD). For comparison between multiple groups, the one-way ANOVA test was performed. For comparison between two groups, the student’s *t* test analysis was performed.

### Study approval

All studies using human subjects were approved by the local ethics committee at Osaka International Cancer Institute (Approval number, No.21003 and No.18010-2) and Nippon Medical School Hospital (Approval number, No.27-02-550). All studies were performed following the Declaration of Helsinki principles.

## Data availability

All data are contained with the manuscript or associated supplementary files.

## Supporting information

This article contains supporting information.

## Conflict of interest

The authors declare no conflicts of interest with the contents of this article.
