# Supplementary material for: A highly specific antibody against the core fucose of the N-glycan in IgG identifies the pulmonary diseases and its regulation by CCL2
Source: J Biol Chem. 2023 Oct 20;299(12):105365. doi: 10.1016/j.jbc.2023.105365 (PMC10663832; doi:10.1016/j.jbc.2023.105365)

**Legends for Supplementary Information**

**Supplementary Figure S1. The recognition of the antibody dose not depend on IgG subclasses.**

**(A)** The intermolecular interaction assay between the antibody against core fucosylated IgG and human IgG_1_, human IgG_2_, human IgG_3_, and human IgG_4_ examined by the Biacore™ system. **(B)** The intermolecular interaction assay between the antibody against core fucosylated IgG and human IgG_1_ treated with or without sodium periodate (NaIO_4_) examined by the Biacore™ system. These all experiments were carried out in duplicate.

**Supplementary Figure S2. The antibody recognizes the core fucose of the *N*-glycan in human IgG.**

**(A)** The binding assay between the antibody against core fucosylated IgG and human IgG_4_ derived from wild type (WT) CHO cells or *Fut8* gene-knockout (*Fut8^-/-^*) CHO cells examined by enzyme-linked immunosorbent assay (ELISA). **(B)** The intermolecular interaction assay between the antibody against core fucosylated IgG and human IgG_4_ derived from wild type (WT) CHO cells or *Fut8* gene-knockout (*Fut8^-/-^*) CHO cells examined by Biacore™ system. **(C)** The intermolecular interaction assay between the antibody against human IgG and human IgG_4_ derived from wild type (WT) CHO cells or *Fut8* gene-knockout (*Fut8^-/-^*) CHO cells examined by Biacore™ system. **(D)** The binding assay between the antibody against core fucosylated IgG and lactoferrin, thyroglobulin, or human IgG purified form human sera examined by ELISA. For **A** and **D**, independent experiments were performed three times and the mean values are shown ± SD.

**Supplementary Figure S3. The recognition of the antibody specific to human IgG.**

**(A)** The binding assay between the antibody against core fucosylated IgG and human IgG, human IgA, human IgM, or rabbit IgG by ELISA. **(B)** The binding assay between the *Lens culinaris* (LCA) lectin (branched Man with Fuc) and human IgG, human IgA, human IgM, or rabbit IgG examined by ELISA. For these data, independent experiments were performed three times and the mean values are shown ± SD..

**Supplementary Figure S4. The antibody can recognize core fucosylated IgG in human serum.**

Western blotting of 10 µl of 1,000 times diluted human pool serum. The core fucosylated IgG and total IgG were analyzed.

**Supplementary Figure S5. Terminal sialylation levels of the *N*-glycan in IgG are not associated with lung diseases**

**(A)** Lectin blotting of IgGs in sera of the patients with lung cancer, COPD, interstitial pneumonia (IP) and control healthy donors. IgGs were purified from the sera with ProteinG/Sepharose and blotted with the *Sambucus sieboldiana* (SSA) lectin (Sia α2,6 Gal), the *Maackia amurensis* (MAM) lectin (Sia α2,3 Gal), and an anti-human IgG antibody. Representative results for three individuals in each disease are shown. **(B)** Normalized band intensities of lectin blotting in **A**. Band intensities were normalized with those of IgG. Lung cancer: N=6, COPD: N=6, IP: N=6, control healthy donors: N=6.

**Supplementary Figure S6. No correlation between KL-6 and the core fucose level of *N*-glycan in IgG in sera of patients with lung diseases**

**(A)** KL-6 level (U/ml) in the sera of patients with lung cancer, COPD, interstitial pneumonia (IP), and control healthy donors examined by ELISA. **(B)** Two-dimensional plot for the level of KL-6 and core fucose of *N*-glycan in IgG in the sera. Lung cancer: N=29, COPD: N=26, IP: N=15, control healthy donors: N=13.

**Supplementary Figure S7. Core fucose of the *N*-glycan in IgG in human B cell lymphoma cell lines**

**(A)** IgG expression in human B cell lymphoma cell lines, SB, Raji, p32/ISH, JY, and Ramos. Total cell lysates (20 µg) were analyzed by Western blotting using an anti-human IgG antibody. Pooled human serum was used as a control. GAPDH was loading control. **(B)** Core fucose of *N*-glycan in IgG in SB and JY cells. IgGs secreted into culture media from SB and JY cells were enriched with ProteinG/Sepharose, a part of them was then analyzed by Western blotting using an antibody against the core fucose of *N*-glycan in IgG (left panel) and an anti-human IgG antibody (right panel). Pooled human serum was used as a control.

**Supplementary Figure S8.** ***FUT8* gene expression in SB and JY cells after incubation with conditioned media prepared from A549 cells**

A549 cells were cultured in Opti-Mem for 3 days, the media then collected as A549 conditioned media, and SB and JY cells were then incubated with this media for an extra 3 days. The *FUT8* gene expressions were examined by RT-qPCR. The expressions were normalized to the *GAPDH* gene.

**Supplementary Table S1. Primers used in this study**

**Experimental procedure for Supplementary Information**

**Enzyme-linked immunosorbent assay (ELISA)**

The 96 well plate (Thermo Fisher Scientific) was coated with 0.25 µg of antigens diluted in PBS by incubation for 2 h at room temperature. Then, the plate was blocked with 1 % (w/v) BSA diluted in 0.05 % Tween 20 in PBS (PBST). After incubation for 2 h at room temperature, 0.5 ng or 1 ng of purified anti-core fucosylated IgG antibody was added. After incubation for 2 h at room temperature, the plate was washed three times with PBST and then incubated with an anti-mouse IgG antibody-labeled with HRP diluted in 0.1 % (w/v) BSA in PBST. After incubation for 1 h at room temperature, the plate was washed three times with PBST and added 50 µl/well of TMB One solution (Promega, Madison, WI). After incubation for 20 min at room temperature, 50 µl/well of 1 N of sulfuric acid to stop the reaction. Finally, the absorbance values at 450 nm were measured with a plate reader.

**Intermolecular interaction assay**

Biacore™ 2000 system (GE Healthcare) was used in this assay. The sensor chip CM5 was first coated with anti-human IgG antibody by using Human Antibody Capture Kit (GE Healthcare) following the manufacturer’s protocol. A 60 µl portion of human IgG_1_, human IgG_2_, human IgG_3_, human IgG_4_, and FLAG-tagged human IgG_4_ derived from wild type CHO cells or *Fut8* gene-knockout CHO cells (50 µg/ml each diluted in HBS-EP buffer: 0.01 M HEPES pH 7.4, 0.15 M NaCl, 3 mM EDTA, 0.005 % w/v Surfactant P20) were then loaded at a speed of 20 µl/min. After loading, the speed was changed to 10 µl/ml and the HBS-EP buffer was flowed for 30 sec. Then, 20 µl of the supernatant of hybridoma producing the anti-core fucosylated IgG antibody was loaded. After loading, the HBS-EP buffer was flowed for 120 sec and monitored the intermolecular interaction of them. For the treatment with sodium periodate (NaIO_4_), 200 µg of IgG in 200 µl of PBS was mixed with 10 µl of 200 mM NaIO_4_ (Wako) and incubated at 4℃ overnight. After the incubation, 200 µl of ethylene glycol (Wako) was added.


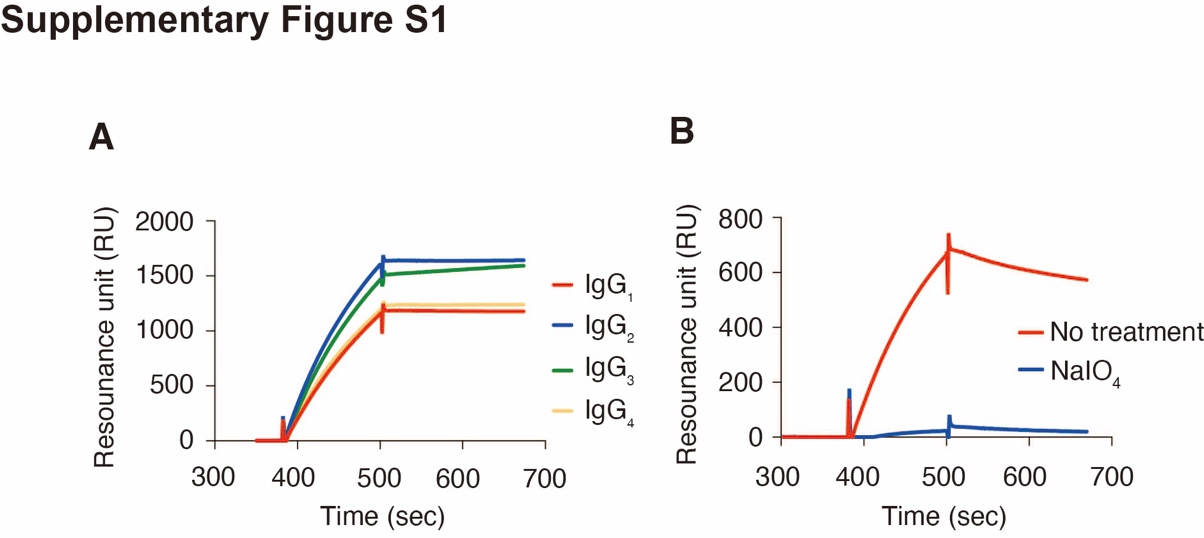


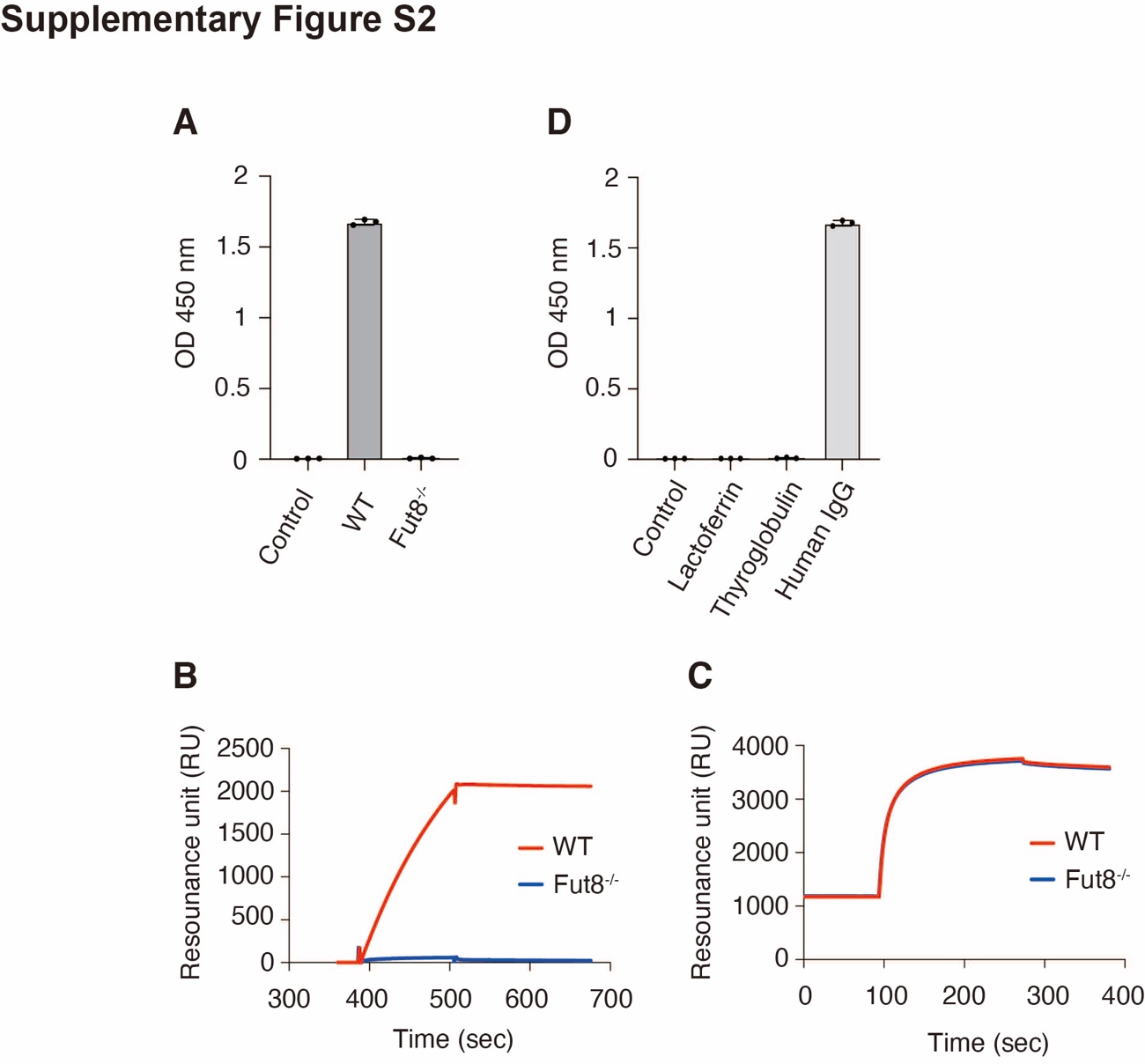


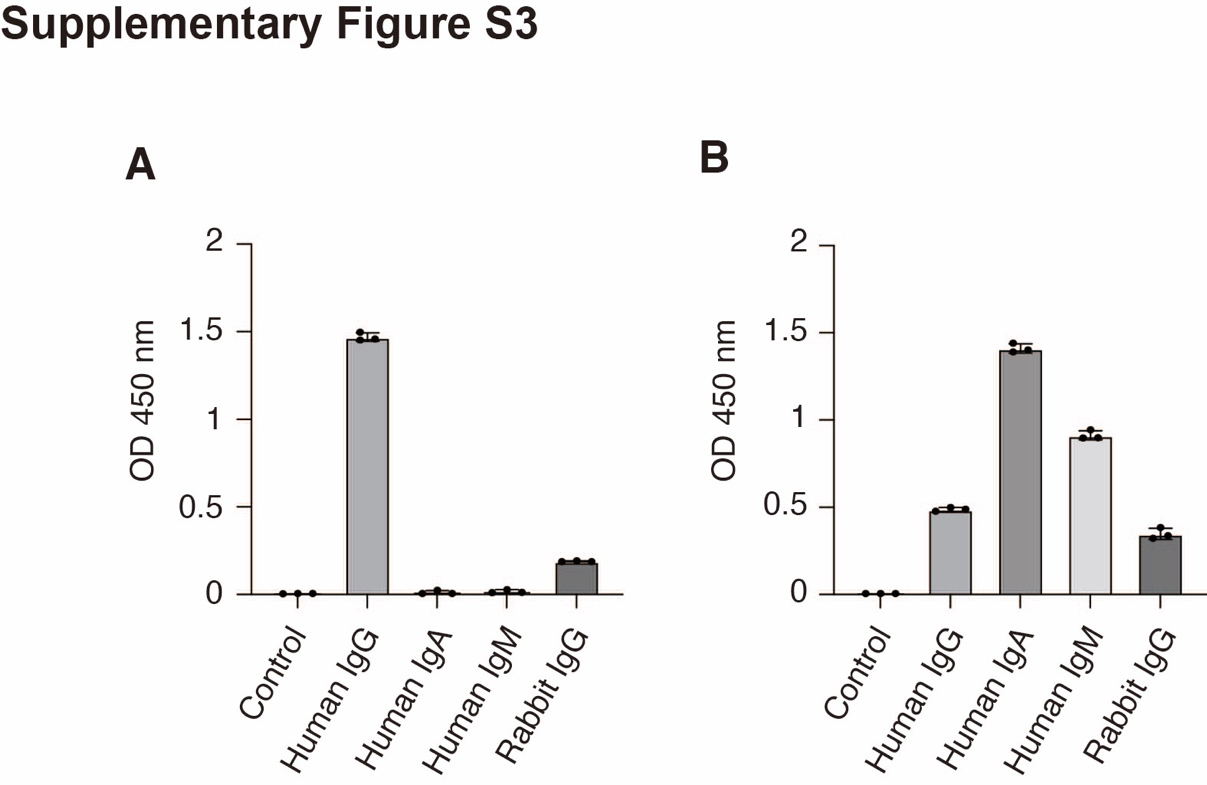


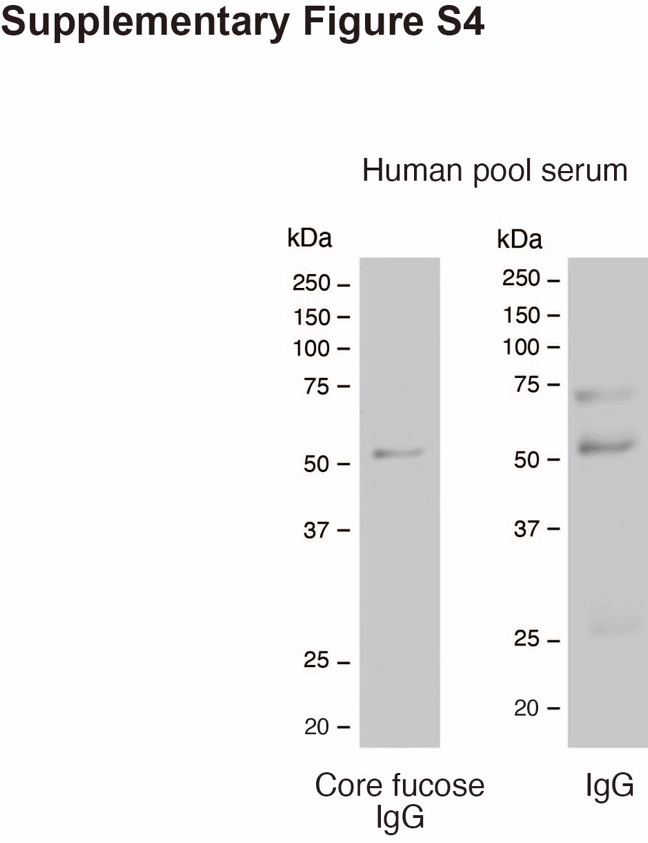


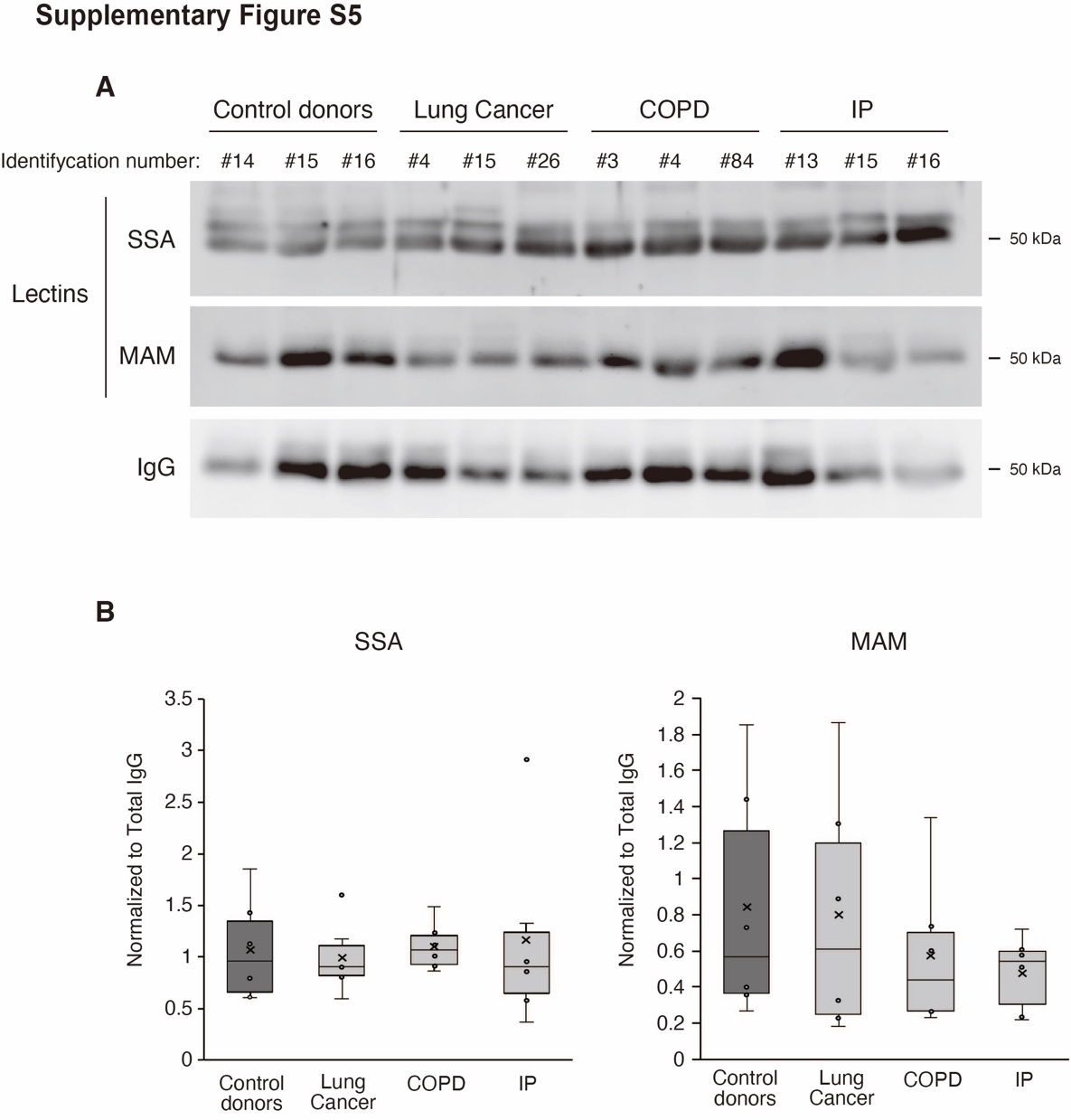


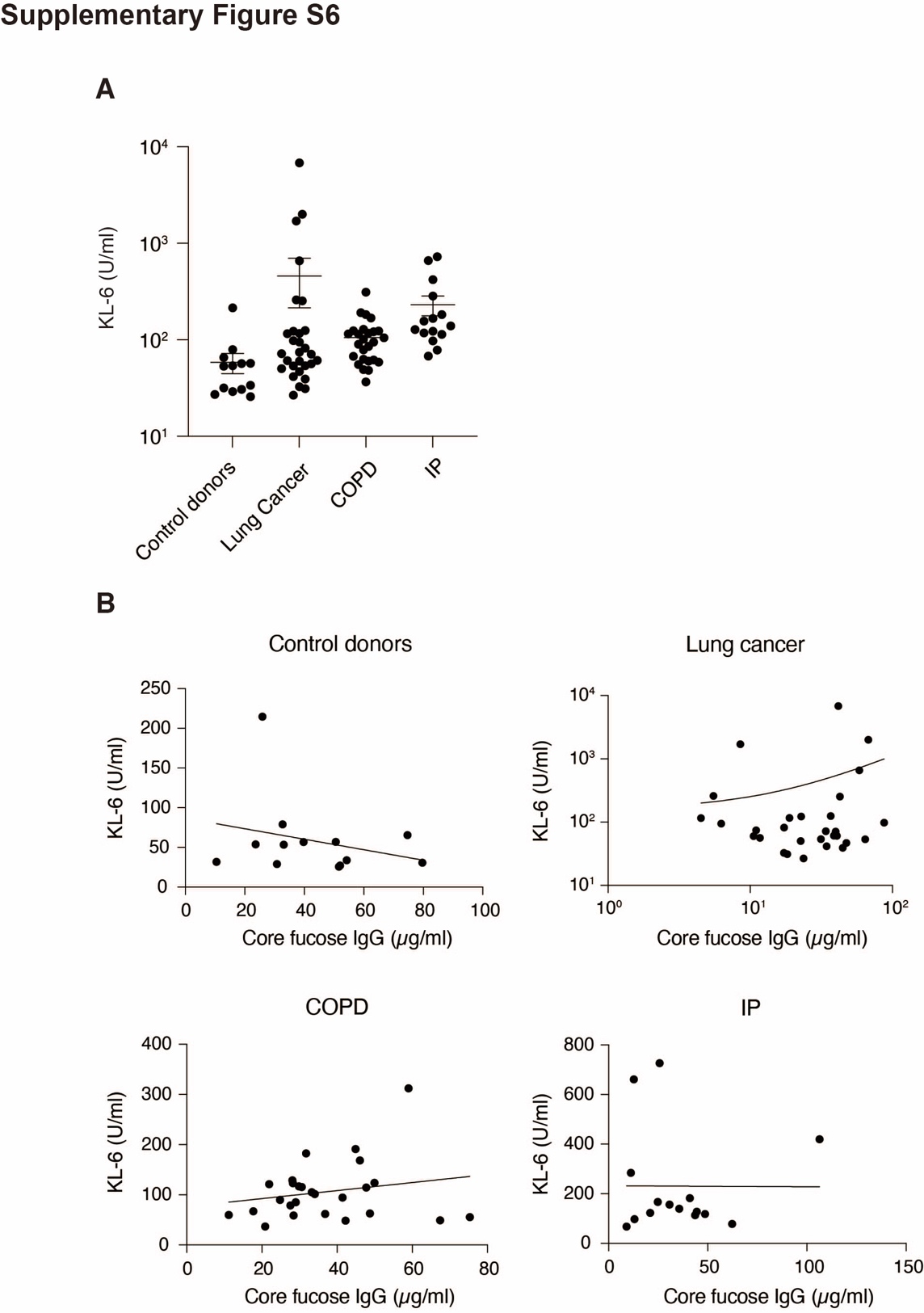

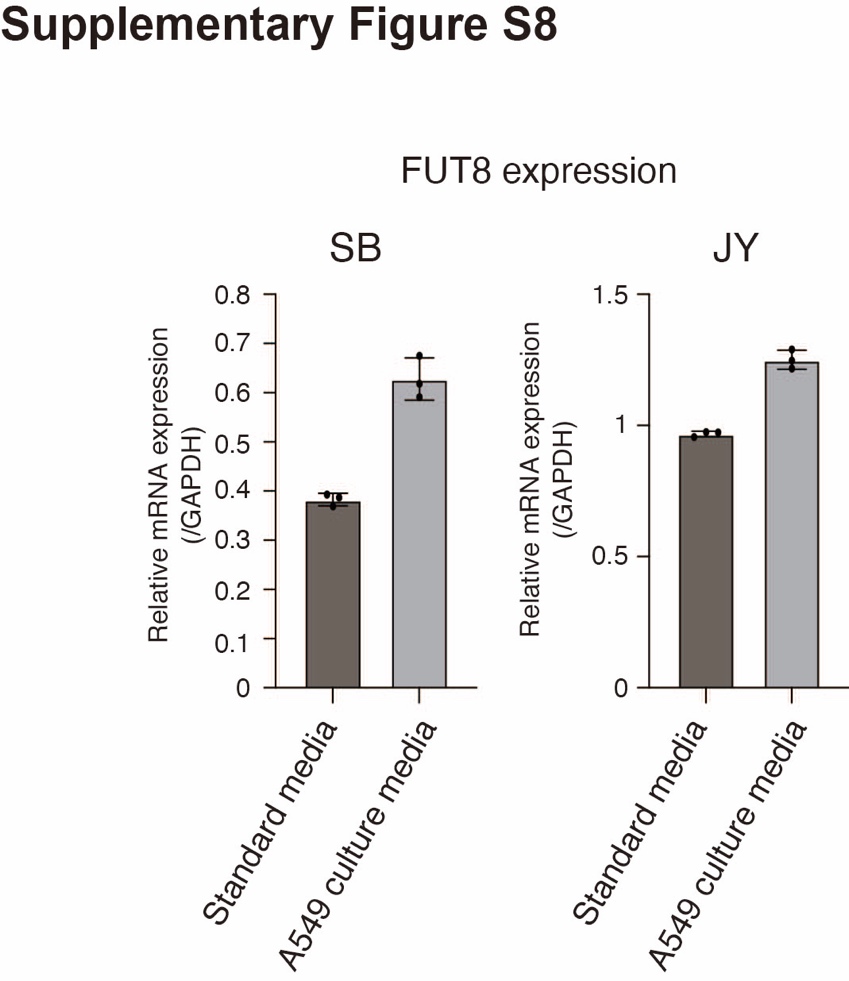

Supplement: Supplementary Figures S1–S8 [file mmc1.docx]
